# An explainable radiomics-based machine learning model for preoperative differentiation of parathyroid carcinoma and atypical tumors on ultrasound: a retrospective diagnostic study

**DOI:** 10.3389/fendo.2025.1617032

**Published:** 2025-08-11

**Authors:** Chunrui Liu, Wenxian Li, Baojie Wen, Haiyan Xue, Yidan Zhang, Shuping Wei, Jinxia Gong, Li Huang, Jian He, Jing Yao, Zhengyang Zhou

**Affiliations:** ^1^ Department of Ultrasound, Nanjing Drum Tower Hospital, Affiliated Hospital of Medical School, Nanjing University, Nanjing, Jiangsu, China; ^2^ Department of Ultrasound, Jinling Hospital, Affiliated Hospital of Medical School, Nanjing University, Nanjing, China; ^3^ Department of Nuclear Medicine, Nanjing Drum Tower Hospital, Affiliated Hospital of Medical School, Nanjing University, Nanjing, Jiangsu, China; ^4^ Department of Radiology, Nanjing Drum Tower Hospital, Affiliated Hospital of Medical School, Nanjing University, Nanjing, Jiangsu, China

**Keywords:** parathyroid neoplasms, parathyroid carcinoma, radiomics, ultrasonography, machine learning

## Abstract

**Background:**

Parathyroid carcinoma (PC) and atypical parathyroid tumors (APT), constituting rare endocrine malignancies, demonstrate overlapping clinical-radiological presentations with benign adenomas. This study aimed to investigate the predictive performance of three radiomics-based machine learning models for the identification of PC/APT from solitary parathyroid lesions using ultrasound.

**Methods:**

This retrospective diagnostic study analyzed 913 surgically-confirmed parathyroid neoplasms (mean age 54.2 ± 13.7 years; 694 females, 219 male) from Nanjing Drum Tower Hospital (n = 730) and Jinling Hospital (n = 183). The cohort comprised 90 malignant lesions and 823 benign adenomas, divided into training (Hospital I) and external test cohort (Hospital II). A radiomic signature derived from 544 quantitative ultrasound features was developed using three machine learning classifiers: Random Forest (RF), Support Vector Machine (SVM), and Logistic Regression (LR). The performance of the predictive models was evaluated based on the pathological diagnosis.

**Results:**

The RF-based radiomics model showed excellent diagnostic performance. The AUC of this model (0.933) was higher than that of SVM (0.900, *P* < 0.05) and LR (0.901, *P* < 0.05). The accuracy, precision, recall, and F1-score of RF model in distinguishing PA from APT/PC were 0.940, 0.683, 0.638 and 0.660. The explainable bar chart, heatmap and Shapley Additive exPlanations (SHAP) values were used to explain and visualize the main predictors of the optimal model.

**Conclusion:**

This radiomics framework provides a promising tool to support doctors in the clinical management of parathyroid lesions.

## 1 Introduction

Parathyroid carcinoma (PC) and atypical parathyroid tumors (APT) are relatively rare infiltrative lesions of primary hyperparathyroidism (PHPT) (1). PC accounts for 0.5–5% (2). The median overall survival from the time of diagnosis of PC is 14.3 years, with 5-year and 10-year survival rates is 78–91% and 60–72%, respectively (3). APT is a newly proposed terminology to replace “atypical parathyroid adenoma” in the WHO 2022 classification update to reflect the uncertain malignant potential of these neoplasms (4). APTs have histological features suspicious for PC but lacking evidence of unequivocal invasion and/or metastasis which are the key morphological features of PC (5). APT are comparatively rare that comprises less than 5% of parathyroid tumors but up to 15% in some studies (4, 6, 7). Although molecular analysis (e.g., CDC73 variants) remains essential for definitive postoperative differentiation (8), the preoperative distinction between APT and PC remains challenging. Some study speculates that APT could represent an early stage of PC (9). Unlike parathyroid adenoma (PA), which can be treated by local parathyroidectomy, *en bloc* resection of the invasive parathyroid tumor should be the preferred treatment approach for APT/PC, particularly during the initial surgical intervention. Hence, accurate preoperative identification of PC/APT can facilitate appropriate surgical resection, which is beneficial for improving patient prognosis (10, 11).

Due to its rarity, there is still no consensus on preoperative identification of typical PA and parathyroid tumors (PC/APT). Patients with PC may present with severe hyperparathyroidism, hypercalcemia, and severe osteoporosis. Nevertheless, in rare cases, PC presents as normocalcemic hyperparathyroidism (12). Due to similar clinical manifestations, some parathyroid tumors are often misdiagnosed as benign parathyroid diseases before surgery. As a result, doctors may mistakenly identify a parathyroid tumor as a less serious parathyroid problem when examining a patient before performing surgery. Preoperative fine needle aspiration (FNA) and intraoperative biopsy are insufficient for diagnosis PC or APT. Moreover, FNA in patients increases the risk of tumor cell seeding along the needle tract (13). Consequently, it is of particular importance to develop non-invasive imaging indicators that can predict the malignant potential of parathyroid lesions prior to the manifestation of serious clinical symptoms.

Ultrasound is the primary imaging modality for hyperparathyroidism, effectively differentiating benign from malignant parathyroid lesions. In the reviewed studies, parathyroid malignant lesions manifest internal heterogeneity differing from benign adenomas, including tumor irregularity and heterogeneity, intratumoral calcification, and parathyroid tumor length exceeding 3 cm (13, 14). Our research has found intact parathyroid hormone (iPTH) (*OR*:1.019), shape (*OR*: 16.625), and relation with the thyroid capsule (*OR*: 3.422) were independent predictive factors associated with the risk of APT/PC (15). Research findings also indicated that DR (two diameters’ ratio of the lesion) and tumor infiltration were independent predictors of malignancy (14). Additionally, emerging ultrasound technologies, such as elastography, provide supplementary diagnostic value in distinguishing adenomas from APT/PC (10, 16). However, ultrasound examinations largely depend on the experience and skill level of the operators, and there may be certain discrepancies in the examination results among different operators, lacking good consistency. As a consequence, the macroscopic visual assessment in ultrasound remains a challenge.

Radiomics, as a quantitative image analysis methodology, has exhibited substantial clinical utility in pathological condition identification, molecular profile classification, and therapeutic outcome prognostication (17, 18). For hand-crafted radiomic, the regions of interest (ROI) are segmented manually by experienced radiologists or experts (19). Feature screening serves to trim down the dimensionality of features, singling out a subset of features that are optimal for the given task. By extracting and analyzing high-dimensional features from imaging data, radiomics can provide more objective and quantitative assessments of parathyroid lesions. This advanced approach could potentially overcome the limitations of traditional ultrasound evaluation, enhance the diagnostic accuracy and reproducibility of ultrasound-based evaluation (20). Zhou et.al (21) developed a machine learning model using high-frequency ultrasound images to differentiate hyper-functioning parathyroid glands in secondary hyperparathyroidism (SHPT) patients. The study used PyRadiomics to extract seven radiomics feature categories, combining them with ultrasound visual features and refining via LASSO regression to select 12 key predictors. Among four machine learning algorithms, the Random Forest (RF)-based model achieved optimal performance (AUC = 0.859). Krupinova et.al (22) developed a mathematical model using CatBoost gradient boosting algorithm based factor such as for the noninvasive preoperative differential diagnosis of PC, APT, and adenoma. To our knowledge, there is limited studies based on ultrasound radiomics for identifying benign and malignant parathyroid lesions.

This study aims to investigate the explainable radiomics models for the preoperative identification of potentially parathyroid tumors in ultrasound.

## 2 Materials and methods

### 2.1 Patients

In this retrospective diagnostic study, a total of 1057 PHPT patients with parathyroid neoplasms who underwent surgical treatment from the two hospitals (Nanjing Drum Tower Hospital and Jinling Hospital) between January 01, 2016 and December 30, 2024 were consecutively enrolled. All patients underwent a standardized dual-modality localization protocol comprising 99mTc-sestamibi SPECT/CT and parathyroid ultrasound. Surgery was indicated only with concurrence of: (1) biochemical confirmation (hypercalcemia + elevated PTH) and (2) positive localization on either SPECT/CT or ultrasound. Cases with discordant/non-localizing imaging underwent further evaluation (e.g., 4D-CT). This retrospective study was approved by the ethics committee of the participating hospital (2024-611-01) and adhered to the principles outlined in the Declaration of Helsinki and Good Clinical Practice guidelines. The requirement for informed consent from patients was waived.

**Figure 1:**
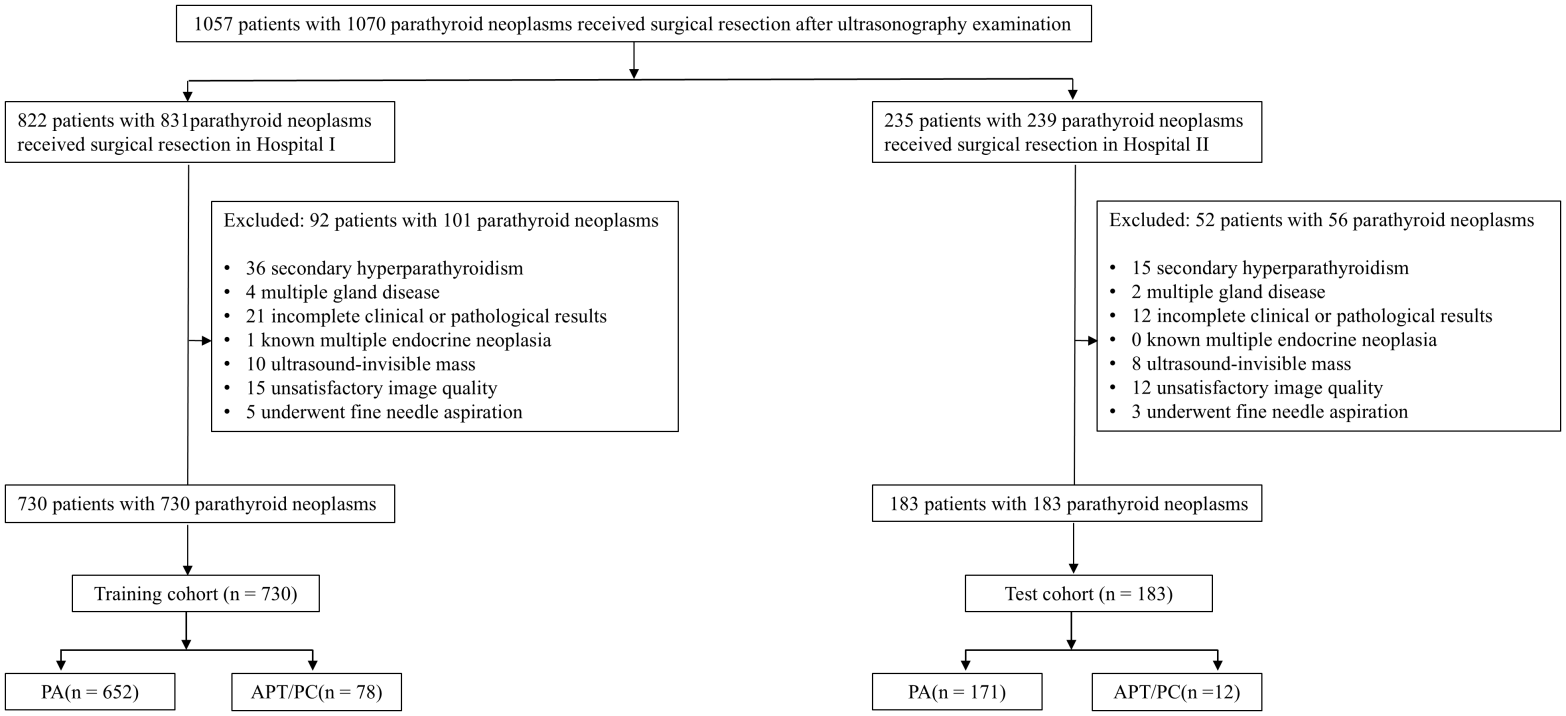
Flowchart of the included subjects. PA, Parathyroid adenoma; APT, Atypical parathyroid tumors; PC, Parathyroid cancer.

**Figure 2:**
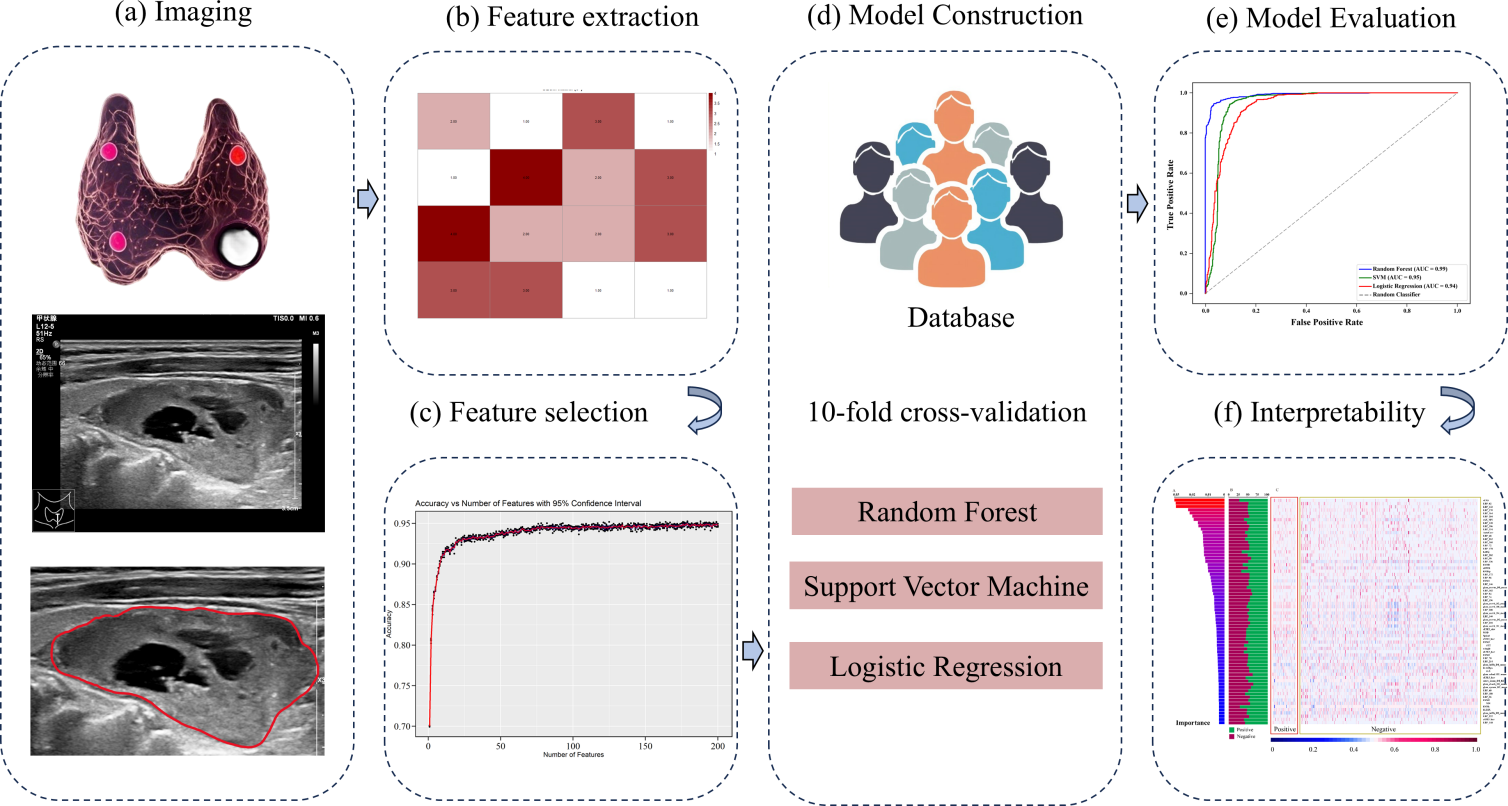
Flowchart of radiomics model proposed in this study. **(a)** Ultrasound image acquisition and Region of Interest (ROI) segmentation of parathyroid lesions. **(b)** Extraction of handcrafted radiomic features. **(c)** Feature selection using statistical methods. **(d)** Model construction employing Random Forest, Support Vector Machine, and Logistic Regression algorithms. **(e)** Model performance evaluation. **(f)** Interpretability analysis: Feature contribution assessment for the optimal model.

Inclusion criteria: 1) preoperative ultrasonographic evaluation conducted within 7 days preceding parathyroidectomy; 2) comprehensive clinical documentation including calcium and phosphate metabolism parameters; 3) histopathological verification per 2022 WHO classification (PA/APT/PC subtypes); 4) minimum 6-month postoperative surveillance. Exclusion criteria:1) secondary hyperparathyroidism or genetic predisposition syndromes (MEN1/2A); 2) incomplete biochemical/imaging records; 3) ambiguous histodiagnosis; 4) suboptimal sonographic visualization preventing lesion characterization; 5) metastatic parathyroid carcinoma; 6) prior fine-needle aspiration potentially altering tissue architecture. The data of the participants were manually obtained from medical records, imaging repositories, as well as pathology findings reports. A flowchart outlining the study design is shown in **Figure 1**. A schematic overview of the study design is illustrated in **Figure 2**.

### 2.2 Image segmentation, feature extraction and selection

The region of interest (ROI) of each parathyroid lesion was segmented on ultrasound images by reader 1 (L. C., with over 7 years of thyroid and parathyroid US interpretation experience) using ImageJ software (http://imagej.net), blinded to pathological outcomes. To assess reproducibility, 60 random selected cases were independently resegmented by both Reader 1 and Reader 2 (X.H., with 10 years of thyroid and parathyroid US interpretation experience) after a 1-month washout period.

Handcrafted features were extracted in MATLAB (vision 2021b) following standard feature extraction protocols (23). The extracted features in this study include computing morphological and texture features. Inter- and intra-observer agreement was quantified using intraclass correlation coefficients (ICC) for both ROI segmentation and feature extraction, with ICC > 0.80 indicating excellent reproducibility, according to Cicchetti’s guidelines.

### 2.3 Model construction and evaluation

To address the data imbalance between APT/PC and PA, a synthetic minority oversampling technique (SMOTE) was applied exclusively to the training dataset after the train-test split, thereby ensuring that no synthetic samples were introduced into the test set and preventing any risk of data leakage (24). Three different machine learning algorithms were employed to establish a binary classification model, i.e., RF, Support Vector Machine (SVM), and Logistic Regression (LR). The radiomics features were used as input to each of these models. All features were standardized prior to model training to ensure uniform scale. Model performance was evaluated using 10-fold cross-validation. In details, the dataset was randomly partitioned into ten equally sized folds. In each iteration, one-fold was reserved as the validation set, while the remaining nine were used for training. This process was repeated ten times to ensure robust performance metrics of model performance. The area under the receiver operating characteristic curve (AUC), accuracy, precision, recall, and the F1 Score were used to assess of the models’ ability to differentiate between PA and APT/PC.

### 2.4 Interpretability

In this case, the RF model yielded the highest AUC, to refine the RF model and identify the most informative features, a stepwise feature selection process was implemented. The process involved the following steps: 1) All features were initially fed into the RF model, and feature importance was ranked using the Gini impurity criterion. 2) Starting from the highest-ranked feature, increasing numbers of features (ranging from 1 to 200) were iteratively fed into the RF model. For each feature subset, 10-fold cross-validation was applied to evaluate model’s accuracy. This process was repeated five times to ensure reproducibility and mitigate the effects of random variability. 3) The accuracy scores for each subset were plotted against the number of features. The feature subset yielding the highest cross-validated accuracy with the smallest number of features was identified to construct the final model. The selected features were standardized to a 0–1 scale to facilitate comparison across samples. A heatmap was generated to visualize the normalized feature set, which provide an intuitive representation of feature patterns across the dataset. The proportion of each feature in these two groups was also calculated to assess its relative prevalence and contribution to the classification process. To enhance model interpretability, significant features were ranked and visualized by SHAP (Shapley Additive exPlanations) values (25).

### 2.5 Statistical analysis

Statistical analysis of basic clinical information was performed using SPSS package (version 23.0). A two-sided chi-square test was performed to determine significant differences in sex between the two groups. Differences in age distribution were evaluated using the student t-test. All model development, performance evaluation, and data visualization were implemented using Python (version 3.8.5). The machine learning algorithms were executed using the scikit-learn library (version 1.3.2), and data visualization, including the heatmap, was generated using Matplotlib (version 3.4.1) and Seaborn (version 0.12.2). AUC, accuracy, precision, recall, and the F1 Score were used for evaluating model performance. All *P* values < 0.05 were considered statistically significant.

## 3 Results

### 3.1 Clinical characteristics

**Table 1** summarizes the clinical parameters and pathological subtypes of 913 patients with parathyroid neoplasms from the two hospitals between January 01, 2016 and December 30, 2024. Overall, 694 (76.0%) patients were female, 219 (24.0%) patients were male, and the mean age was 54.2 ± 13.7 years. Ninety (9.9%) malignant lesions of the 913 lesions were PC (n = 3) and APT (n = 87), while 823 (90.1%) lesions were benign adenomas. The patients were classified into a training cohort (n = 730) and a test cohort (n = 183), respectively. The rates of APT/PC in the training and test cohorts (10.7% and 6.6%, respectively) were not significantly different (*P* = 0.098). The serum iPTH showed a significant difference between the training and test cohorts (*P* = 0.015), and other indicators showed no difference between the two groups (*P* > 0.05).

**Table 1:** Baseline characteristics of study sets.

Characteristic	Total (n = 913)	Training cohort (n = 730)	Test cohort (n = 183)	*P* value
Sex, n (%)				0.323
Male	219 (24.0%)	170 (23.3%)	49 (22.4%)	
Female	694 (76.0%)	560 (80.7%)	132 (19.3%)	
Age at diagnosis, yr	54.2 ± 13.7	54.3 ± 14.0	53.8 ± 12.5	0.312
serum iPTH, pmol/L [M (Q_1_, Q_3_)]	17.3 (11.9,23.6)	19.0 (8.0,22.6)	16.1 (9.6,22.8)	0.015
serum calcium, mmol/L [M (Q_1_, Q_3_)]	2.6 (2.5,3.0)	2.6 (2.5,2.9)	2.8 (2.5,2.9)	0.066
serum phosphate, mmol/L [M (Q_1_, Q_3_)]	0.5 (0.6,0.9)	0.5 (0.6,0.9)	0.5 (0.3,0.9)	0.167
Pathological subtype				0.098
PA	823 (90.1%)	652 (89.3%)	171 (93.4%)	
APT/PC	90 (9.9%)	78 (10.7%)	12 (6.6%)	

PA, Parathyroid adenoma; APT, Atypical parathyroid tumors; PC, Parathyroid cancer.

### 3.2 Model performance based on radiomics

**Figure 3:**
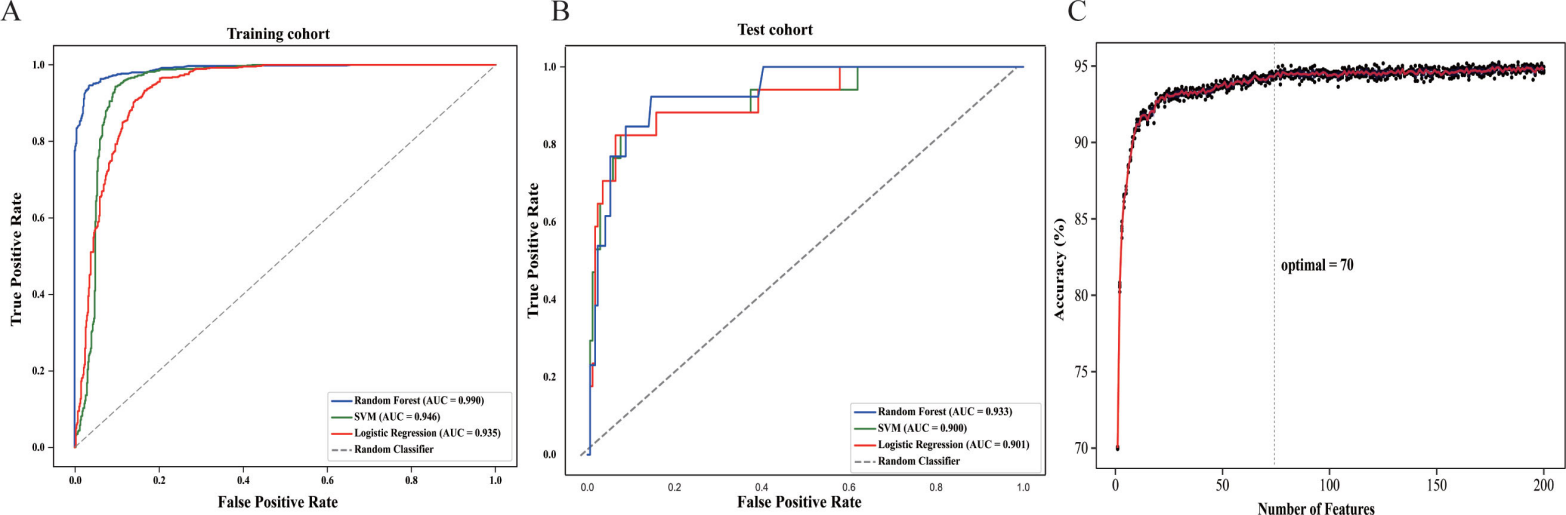
The construction of the radiomics model **(A)** In the training cohort, the area under the receiver operating characteristic curve (AUC) of Random Forest (RF), Support Vector Machine (SVM), and Logistic Regression (LR) models were 0.990, 0.946 and 0.935, respectively. **(B)** In the test cohort, the AUC of RF, SVM, and LR models were 0.933, 0.900 and 0.901, respectively. **(C)** The accuracy-feature number plot showed that the top 70 features were sufficient to build an optimal model without significant gains from adding additional features.

A total of 544 radiomic features were extracted from each ultrasound image. All the radiomic features with high reproducibility and stability (ICC > 0.80). Three machine learning models including RF, SVM, and LR were evaluated for their performance based on the AUC (**Figures 3A, B**). **Table 2** summarizes the predictive performance of radiomic models for parathyroid tumor estimation across training and test cohorts. In the training cohort, the RF model had the highest predictive performance in the test cohort, with an AUC of 0.933, higher than that of SVM (0.900, P < 0.05) and LR (0.901, P < 0.05). Its accuracy, precision, recall, and F1-score for distinguishing PA from APT/PC were 0.940, 0.683, 0.638, and 0.660. SVM and LR had lower performance metrics compared to RF. RF’s accuracy, precision, and F1-score were statistically better than SVM’s (P < 0.05). While LR had the highest recall (0.770) in the test group, its precision was only 0.345.

**Table 2:** The prediction power of the radiomic model for estimating parathyroid tumors.

Cohort	Model	AUC	Accuracy	Precision	Recall	F1-score
Training cohort	Random Forest	0.990^*^‡	0.952^*^‡	0.952^*^‡	0.951^*^	0.952^*^‡
	Support Vector Machine	0.946	0.896	0.838	0.983‡	0.905
	Logistic Regression	0.935	0.878	0.827	0.956	0.887
Test cohort	Random Forest	0.933^*^‡	0.940^*^‡	0.683^*^‡	0.638	0.660^*^‡
	Support Vector Machine	0.900	0.848	0.286	0.769	0.417
	Logistic Regression	0.901	0.880	0.345	0.770‡	0.476

AUC area under the receiver operator characteristics curve; ^*^ *P* < 0.05; ‡, the highest metric among all three models.

According to the accuracy-feature number plot, the model’s accuracy was 0.70 when using only one feature. As additional features were included, the accuracy steadily improved, reaching a plateau around 0.93 when approximately 70 features were used (**Figure 3C**). Hence, the top 70 features (as described in **Supplementary Table S1**) were sufficient to build an optimal model without significant gains from adding additional features.

**Figure 4:**
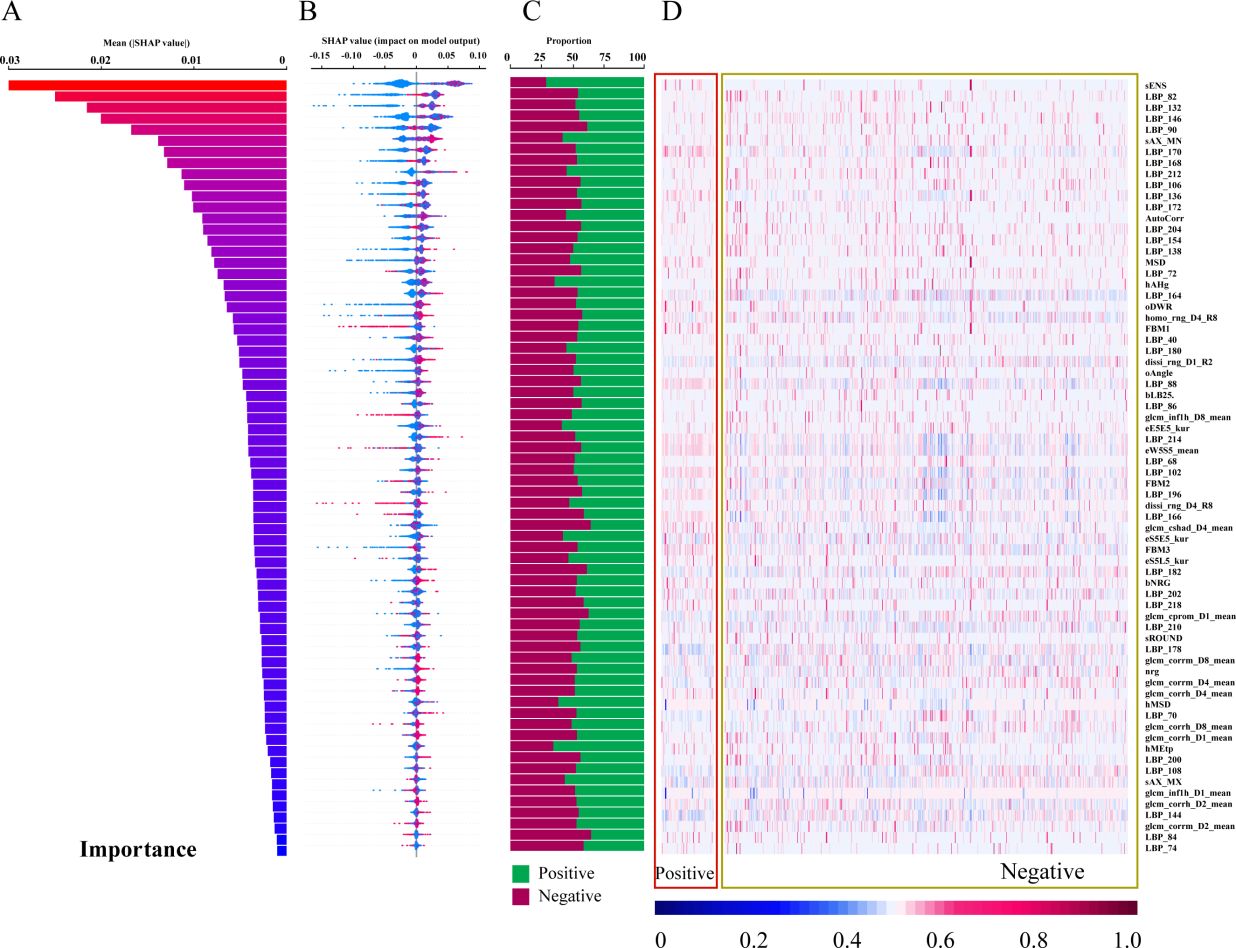
Feature contribution analysis for the Random Forest Model. **(A)** Feature importance scores ranked in descending order (top 70 features shown). **(B)** SHAP values for each feature, ranked by descending importance. **(C)** Bar chart visualizing feature importance. **(D)** Heatmap visualizing feature contributions.

The diagram of the 70 most important features contributing to Random Forest were shown in **Figure 4**. The 70 important features are ranked by relative importance scores and shown in **Figure 4A**. The feature importance ranked in descending order is presented in **Figure 4A**, which illustrates the relative importance of each feature. Specifically, sENS is the most influential feature. LBP_XX features are commonly found among the top 70 features. sAX_MN, AutoCorr, hAHg, and hMSD are also important features. SHAP was used to calculate their individual contributions to the model’s predictions (**Figure 4B**). A positive SHAP value signifies a positive association with the model output, whereas a negative value indicates a negative association. The distribution of features was then visualized using a heatmap, and the average standardized values were calculated through statistical analysis (**Figures 4C, D**). Most features exhibited proportions around 50 ± 10%, with only five features (sENS, hAHg, hMSD, hMEtp and hMSk) showing a preference of approximately 70%.

## 4 Discussion

In this study, we developed an explainable radiomics model derived from parathyroid sonographic images to accurately diagnose APT/PC in PHPT patients. Three different ML classifiers were initially applied, and the RF classifier was shown to outperform others in both training and testing datasets. Regarding the interpretation of selected features, the 70 important features ranked by relative importance scores revealed that sENS and LBP_XX had a greater impact on identifying APT/PC. The constructed model provides a cost-effective tool for assessing potentially parathyroid tumors that can intelligently provide guidance to surgical strategy and long-term monitoring.

Firstly, this study established a radiomics-based RF model with high accuracy in distinguishing parathyroid adenomas from neoplastic lesions. The RF model achieved higher AUC than LR and SVM models, attributed to its ability to capture complex, non-linear relationships between features. We visualized the top 70 features contributing to the RF model, representing diverse radiomic characteristics including intensity-based, shape-based, and texture-based descriptors. sENS, representing texture intensity of small image areas, is the most influential feature, indicating image intensity-based values correspond to underlying physiological properties of the tissue. LBP_XX features, extracted by the Local Binary Pattern operator, are common among the top 70 features and represent local textural information by comparing pixel grayscale values with surrounding neighborhoods (26). sAX_MN is a shape-based characteristic. hAHg and hMSD are GLCM-based features. AutoCorr, the autocorrelation feature, quantifies the correlation between pixel values in an image and describes repetitive patterns and periodicity of textures. These radiomic features, assessing spatial relationships between voxel intensities within a region of interest or between voxels and their surroundings, may indicate varying patterns of heterogeneity in parathyroid masses. This addresses the poor interpretability of “black-box” nature of AI models.

In our study, the importance of each feature in our study was visualized in the output of heatmap, where the majority of features exhibited proportions around 50 ± 10%, with only five features (sENS, hAHg, hMSD, hMEtp, and hMSk) showing a preference of approximately 70%. This highlights the intrinsic complexity and multifactorial nature of tumor classification, where no single feature provides a decisive binary classification. Instead, the radiomic data unveils a nuanced pattern of contributions, where the interplay of multiple features influences the model’s prediction. Even the most distinguishing features showed only a modest preference for positive or negative contributions, reinforcing the notion that tumor classification relies on an integrated assessment of diverse characteristics. This complexity mirrors the biological heterogeneity of tumors, which often exhibit varying textures, shapes, and intensities across different regions and between different samples. The variability within and between tumor samples underscores the necessity of leveraging comprehensive radiomic analyses, rather than isolating individual features, to capture the full spectrum of tumor characteristics. Furthermore, the SHAP framework interprets RF models by quantifying feature contributions to predictions, where the higher the SHAP value of the feature, the stronger the correlation of parathyroid pathological classification. This method identifies key predictors and their impact on outcomes, enhancing model transparency in clinical decision-support systems. To sum up, this multi-dimensional explainable approach aligns with the clinical understanding of tumor biology, where no single imaging characteristic can accurately capture the full complexity of tumor behavior. Thus, the integration of multiple imaging features through advanced radiomics offers a more robust and reproducible diagnostic framework, paving the way for more precise clinical decision-making.

Although radiomics and machine learning have been widely used in ultrasound image analysis and disease prediction, few research reports exist on their application in diagnosing parathyroid cancer. Valavi et al. (27) investigated radiomics-based differentiation of parathyroid adenomas from normal tissue using delayed-phase SPECT/CT scans in 92 patients (58 adenomas, 34 normal). After extracting 65 radiomic features, three selection methods (MRMR, RFE, Boruta) were combined with six machine learning models. The RFE+XGB combination achieved peak AUC (0.76 ± 0.08), while MRMR+GB showed optimal accuracy (72 ± 7.2%). Sensitivity and specificity maxima were attained through RFE+SVM (94 ± 5.5%) and Boruta+SVM (82 ± 12%), respectively. Yeh and colleagues (28) developed a novel machine learning algorithm (MLCDA) utilizing random forest to localize 458 hyperfunctioning parathyroid glands via 4D-CT/MIBI SPECT/CT in PHPT patients. The model identified three critical predictors: 4D-CT/MIBI sensitivity, specificity, and calcium × PTH product, achieving 91% training and 90% validation accuracy across five probability categories. To our knowledge, this represents the first investigation employing ultrasound radiomics for preoperative differentiation of APT/PC.

There are several limitations in this study. Firstly, this was a retrospective analysis, and prospective multicenter cases are needed to confirm our findings. Secondly, the cohort exhibited marked class imbalance (90 APT/PC vs. 823 PA cases), a clinically ubiquitous phenomenon in parathyroid lesion studies. This imbalance, while reflecting real-world disease prevalence patterns, poses inherent challenges for radiomics-based AI models through potential majority-class bias amplification. Hence, we used SMOTE to address the data imbalance problem in radiomics (24). Thirdly, the models depend on specific features and data partitioning methodologies chosen by our researchers. These models are not fully autonomous, requiring human intervention in their design. Their performance is influenced by design choices made during development. While these models may show efficacy when applied to data used in their creation (retrospective data), their performance may be suboptimal when applied to novel, external datasets. Fourthly, the feature importance plot in our study only reflects the magnitude of feature importance and fail to distinguish the specific directional impact of features on prediction outcomes, necessitating additional manual labeling of positive/negative influences. These constraints reduce their reliability for medical image analysis.

In conclusion, the system developed offers a promising tool to support doctors in managing parathyroid lesions clinically. Timely identification of potentially malignant parathyroid tumors and subsequent surgical intervention are of considerable clinical significance. To enhance the model’s clinical applicability, future investigations should explore the integration of radiomics with clinical decision-making tools, such as biomarkers like iPTH and calcium levels. Additionally, incorporating clinical and demographic predictors into the decision-making process could further improve diagnostic accuracy and provide a more comprehensive approach to patient management.

## Data Availability

The raw data supporting the conclusions of this article will be made available by the authors, without undue reservation.
