# Supplementary material for: An explainable radiomics-based machine learning model for preoperative differentiation of parathyroid carcinoma and atypical tumors on ultrasound: a retrospective diagnostic study
Source: Front Endocrinol (Lausanne). 2025 Aug 11;16:1617032. doi: 10.3389/fendo.2025.1617032 (PMC12375457; doi:10.3389/fendo.2025.1617032)
Supplement: Supplementary file 1 [file Table1.docx]

Supplement table 1 top 70 features predictions for parathyroid tumors based on ultrasound radiomics

| TOP 1-10 | TOP 11-20 | TOP 21-30 | TOP 31-40 | TOP 41-50 | TOP 51-60 | TOP 61-70 |
| --- | --- | --- | --- | --- | --- | --- |
| sENS | AutoCorr | hMSD | LBP_74 | eE5E5_skw | LBP_214 | LBP_180 |
| LBP_82 | LBP_68 | oDWR | LBP_196 | MSD | glcm_inf1h_D4_mean | LBP_36 |
| LBP_132 | LBP_212 | hMEtp | glcm_corrh_D2_mean | Spicul | bLB10px | FBM5 |
| LBP_170 | LBP_168 | LBP_172 | glcm_corrh_D8_mean | eS5L5_kur | sLS | hSk |
| LBP_164 | LBP_72 | LBP_86 | LBP_200 | FBM3 | glcm_cshad_D2_mean | hMSk |
| LBP_204 | LBP_178 | FBM1 | glcm_corrh_D4_mean | sSC | eE5L5_kur | bLB25. |
| sAX_MN | hAHg | LBP_146 | LBP_140 | oAngle | entro_mean_D4_R2 | glcm_inf1h_D8_mean |
| LBP_138 | LBP_202 | glcm_corrm_D8_mean | glcm_corrm_D2_mean | eE5E5_kur | glcm_dvarh_D1_mean | LBP_222 |
| LBP_106 | LBP_90 | LBP_182 | LBP_218 | FBM2 | glcm_cprom_D2_mean | eS5E5_kur |
| LBP_154 | LBP_136 | LBP_84 | glcm_corrh_D1_mean | LBP_70 | LBP_40 | LBP_148 |
